# Callous‐unemotional traits and impulsivity: distinct longitudinal relations with mind‐mindedness and understanding of others

**DOI:** 10.1111/jcpp.12445

**Published:** 2015-07-14

**Authors:** Luna C.M. Centifanti, Elizabeth Meins, Charles Fernyhough

**Affiliations:** ^1^ Department of PsychologyUniversity of DurhamDurhamUK; ^2^ Department of PsychologyUniversity of YorkYorkUK

**Keywords:** Emotion understanding, callous‐unemotional traits, theory of mind, mind‐mindedness, longitudinal

## Abstract

**Background:**

Problems in understanding other people's mental states may relate to distinct personality traits that are associated with early externalizing behavior. A distinction between theory of mind (ToM) and empathy has proven important in shedding light on the problems in understanding other minds encountered by children high on callous‐unemotional (CU) traits and exhibiting impulsivity. The aim of this study was to investigate whether children's early ToM and emotion understanding abilities predicted CU traits and impulsivity at age 10. A further aim was to explore whether the quality of the parent–child relationship very early in the development indirectly or directly predicted the children's CU traits and impulsivity.

**Method:**

We examined whether ToM and empathy skills might differentially relate to personality traits associated with externalizing behaviors (i.e., impulsivity and CU traits). We examined these relations over time in a longitudinal cohort of 96 boys and girls using follow‐back analyses, incorporating measures of maternal mind‐mindedness (appropriate mind‐related talk) to examine the possible role of parent–child interaction quality.

**Results:**

Appropriate mind‐related talk indirectly predicted CU traits (at age 10 years) via its effect on children's emotion understanding. ToM predicted impulsive/irresponsible traits, but ceased to be significant when controlling for externalizing behaviors.

**Conclusion:**

The present findings demonstrate that parents who remark appropriately on their infant's mental states may help the child to understand emotions and may mold an empathic understanding of others, thereby preventing CU traits.

## Introduction

Theory of mind (ToM), emotion understanding, and empathy are different facets of understanding other people's mental states. ToM requires an understanding of others' mental perspectives, whereas emotion understanding indexes the ability to recognize and label emotional expressions and appreciate how certain situations give rise to emotional reactions. While empathy additionally requires the ability to recognize how others would feel as a result of their different perspectives (Völlm et al., 2006), tasks typically used to assess emotion understanding (e.g., Denham, 1986) include a measure of the children's recognition that others may react differently from them in a particular situation.

Poor ToM abilities relate to attention and impulsivity/hyperactivity problems (Fahie & Symons, 2003; Perner, Kain, & Barchfeld, 2002), which in turn are associated with the loss of control and aggression in response to provocation from peers (see Muñoz & Frick, 2012; for a review), and perceiving hostile intent in other people's actions even when these actions are ambiguous (Marsee & Frick, 2007). ToM deficits are not, however, seen in all children who present with difficult behavior. The distinction between mentalistic and affective perspective‐taking has proved particularly important in shedding light on the problems in understanding other minds encountered by children with callous‐unemotional (CU) traits. CU traits include a lack of caring for values that others share, a lack of remorse, and a general poverty of affect, and have been found to relate to high rates of aggression and externalizing behaviors (Muñoz & Frick, 2012). Children high on CU traits have intact ToM (O'Nions et al., 2014) but encounter problems in emotion processing (Muñoz, 2009; Sharp, Vanwoerden, Van Baardewijk, Tackett, & Stegge, 2015) and empathy, as indexed by both self‐ and parent‐report (Dadds et al., 2009; Muñoz, Qualter, & Padgett, 2011). Moreover, when such children are aggressive, their aggression tends to be cold and unemotional and perpetrated for personal gain (Marsee et al., 2014). This specific deficit in affective perspective‐taking led Dadds et al. (2009) to describe youth with CU traits as being able only to ‘talk the talk’ of emotions.

However, although these studies suggest poor empathy is characteristic of children with CU traits, they assessed empathy using self‐ or parent‐report, rather than observational or task‐based assessment. Consequently, no study has yet directly investigated the link between CU traits and children's emotion perspective‐taking. In addition, CU traits or impulsivity/hyperactivity were assessed concurrently with ToM or empathy, so these studies do not speak to the issue of whether difficulties in understanding others' mental or emotional states early in development predict CU traits. Finally, no study has investigated whether children with impulsivity/hyperactivity problems have intact emotion understanding, so a double dissociation has not been established. The aim of this study was, thus, to investigate whether the children's performance on ToM and emotion understanding tasks early in development predicted CU traits and impulsivity at age 10. Given the findings discussed above for concurrent relations between CU traits and deficits in empathy, and between impulsivity and ToM, we investigated whether (a) CU traits at age 10 were predicted by poorer emotion understanding in early childhood, and (b) impulsivity at age 10 was predicted by poorer ToM performance in early childhood.

A further aim of this study was to explore whether the quality of the parent–child relationship very early in development predicted the children's CU traits and impulsivity at age 10. Parents' attunement to their infants' emotions and cognitions is known to predict children's later ToM and emotion understanding. For example, mind‐mindedness indexes the caregivers' tendency to comment appropriately on the infant's putative thoughts and feelings in the first year of life (Meins et al., 2012), and is the earliest identified predictor of ToM and emotion understanding abilities (Laranjo, Bernier, Meins, & Carlson, 2010; Meins, Fernyhough, Arnott, Leekam, & de Rosnay, 2013; Meins et al., 2002). Such appropriate mind‐related comments also predicted lower levels of externalizing behaviors in children growing up in low socioeconomic circumstances (Meins, Centifanti, Fernyhough, & Fishburn, 2013). Thus, appropriate mind‐related comments in the first year appear to play a role in facilitating children's later ToM and emotion understanding, and protect vulnerable children against behavioral difficulties.

Viding, McCrory, and Seara‐Cardoso (2014) suggest that back‐and‐forth mirroring of emotions between parent and infant may lay the foundation for emotion understanding and prevent the development of CU traits, but little research has investigated relations between early parent–infant interaction and later CU traits. One exception is Bedford, Pickles, Sharp, Wright, and Hill's (2015) study, which showed that higher CU traits at age 2 were predicted by lower maternal sensitivity scores at age 29 weeks, but this relation was observed only in girls. No study has investigated how early infant–parent interaction relates to CU traits in later childhood. Moreover, research is virtually silent on the mechanisms via which these early parent–child experiences might shape CU behaviors. Parental responsiveness promotes the children's knowledge and concern about others, and allows for the internalization of parental moral and rule‐based values in early childhood (Kochanska, 1993). Children's early emotion understanding may thus mediate the relation between parent–infant interaction and children's CU traits.

This study investigated this possibility in relation to early mind‐mindedness and general maternal sensitivity (Ainsworth, Bell, & Stayton, 1974). Given the previously discussed relations between mind‐mindedness and children's understanding of mind, we expected mind‐mindedness to play a more important role than sensitivity in predicting the children's later CU traits. Thus, we tested the idea that CU traits at age 10 years are associated with early appropriate mind‐related comments indirectly via emotion understanding. We expected this mediation would remain even when controlling for externalizing behaviors, as the CU traits reflect characteristics that are not synonymous with externalizing behaviors. We tested emotion understanding as a general index of emotion processing across the basic emotions. Although a meta‐analysis conducted by Marsh and Blair (2008) reported significant deficits specifically for fearful faces within antisocial samples, a recent meta‐analysis with community and clinical samples showed deficits across both positive and negative emotions, suggestive of a general rather than specific emotion processing impairment (Dawel, O'Kearney, McKone, & Palermo, 2012). In contrast to CU traits, we expected impulsivity/irresponsibility to be associated with early appropriate mind‐related comments indirectly via ToM. We expected this might be attenuated when including externalizing behaviors as impulsivity is closely linked to a lack of self‐control. In addition, to examine whether the prediction of CU was specific to the mothers' attunement to their infants' internal states, rather than their more general responsivity, we controlled for sensitivity.

## Method

### Participants and procedure

Participants were a sample of 206 mothers and children (108 girls). Potential participants were identified by general practice surgeries and health visitors, and information about the study and an invitation to participate was sent through the mail. Details of mothers who were interested in taking part were passed on to the researchers, and participants were recruited by telephone. Participants were also recruited in person through invited visits to the community mother‐and‐baby groups held in a variety of locations (e.g., church halls, community centers). The vast majority of the mothers who consented to take part (*n *=* *203) were White, and 86 infants were first‐born. Participants came from wide‐ranging socioeconomic status (SES) backgrounds as assessed using the Hollingshead Index (Hollingshead, 1975), with scores ranging from 11 to 66; around half of the sample (*n *=* *90) were from low SES backgrounds (falling into the lowest two Hollingshead categories).

Children's ages at the testing phases were as follows: Phase 1, 8 months (*M *=* *8.52, *SD *= 0.48, range 7.0–10.2); Phase 2, 51 months (*N *=* *161, *M *=* *51.53, *SD *= 0.85, range 49.00–53.00); Phase 3, 61 months (*N *=* *164, *M *=* *61.35, *SD *= 1.08, range 58–64); Phase 4, 10 years (*N *=* *96; Mean =10.3 years, range = 10.1–10.7 years). Attrition was due to families either moving away from the area or being unable to schedule convenient testing times. Those who dropped out at a later phase had lower SES (dropouts: *M* = 26.72, *SD* = 12.45; nondropouts: *M* = 35.70, *SD* = 13.86; Cohen's *d *=* *0.66), but did not differ on any other measures. However, despite this SES‐specific attrition, the sample remained socially diverse, with 32% families in the low SES group at Phase 4.

The study received ethical approval from the relevant University committee and the Ethics Committees of the individual National Health Service Authorities.

### Overview of testing phases

At Phase 1 (8 months), maternal mind‐mindedness and sensitivity were assessed. Children's ToM, emotion understanding, and receptive verbal ability were assessed at Phase 2 (51 months), and parents and teachers reported on children's behavioral difficulties at Phase 3 (61 months). Children's CU traits were assessed at Phase 4 (age 10).

### Measures

#### Mind‐related comments

At Phase 1, mothers and their 8‐month‐olds were filmed in a 20‐min free‐play interaction. Mothers' speech during the interaction was later transcribed verbatim, and all comments which included an internal‐state term referring to the infant's internal state (mind‐related comments) or where the mother spoke in the first person on the infant's behalf were identified from the transcripts. Each mind‐related comment was then coded as appropriate or nonattuned by watching the filmed interaction. A comment was coded as appropriate if any of the following criteria were met: (a) the independent coder agreed with the mother's reading of her infant's mind; (b) it linked the infant's current activity with related past or future events; or (c) it was a suggestion for a new activity after a lull in the interaction (e.g., ‘You'll want to play with this’). Scores were expressed as a percentage of the total number of comments to control for verbosity. Mind‐related comments were coded by a researcher who was blind to all measures and to the hypotheses of the study, with a second blind researcher coding a randomly selected 25% of the interactions. Inter‐rater agreement was *κ * =  .70 (87% agreement).

#### Maternal sensitivity

Ainsworth et al.'s (1974) maternal sensitivity scale was used to code the Phase 1 interactions. Mothers received a score ranging from ‘highly insensitive’ (1) to ‘highly sensitive’ (9). A trained researcher, blind to all other measures and to the study's hypotheses, coded all sessions; a second trained, blind researcher coded a randomly selected 25% of the interactions. Neither researcher was involved coding mind‐mindedness. Inter‐rater reliability (intraclass correlation) was .83.

#### Theory of mind

At Phase 2 (51 months), children completed a battery of ToM tasks based on Wellman and Liu (2004): (a) diverse beliefs, (b) the relation between knowledge and access to information, (c) the relation between the appearance of a container and one's belief about its contents (for both self and other), (d) explicit false belief, and (e) predicting a protagonist's behavior on the basis of his/her false belief. Children, additionally, had to pass all relevant memory and reality control questions for each item to be credited with a correct response. Potential scores ranged from 0 to 6. The ToM battery had adequate internal reliability, *α*=.63, and was at a level similar to prior studies (e.g., Astington & Jenkins, 1999).

#### Children's emotion understanding

At Phase 2 (51 months)*,* Denham's (1986) task and three items from the Test of Emotion Comprehension (TEC) (Pons, Harris, & de Rosnay, 2004) were administered. In Denham's task, the experimenter gives tone‐of‐voice cues to the correct emotional response, but all the TEC items are given in an emotionally neutral tone.

Denham's task consists of three sections: (a) labeling four emotional facial expressions (happy, sad, angry (‘cross’), scared), (b) using the situational context as a cue to the four emotions, and (c) recognizing that people may vary in their emotional responses to the same event. For (a), children were assessed for their ability to both generate the emotional label and to choose the correct face to match the emotional label given by the experimenter. Children received two points for each correct response, one point for an incorrect response of the correct valence (e.g., sad for scared), and zero for an incorrect response. Potential scores ranged between 0 and 16. For (b), children heard four vignettes in which one of the four emotions would unequivocally be felt by the story protagonist (e.g., feeling scared after a nightmare). Children labeled the emotion in each vignette, scoring between 0 and 2 as described above (range 0–8). For (c), the children's mothers had previously reported how they responded to a number of emotionally equivocal situations (e.g., being approached by a dog). The six vignettes in section (c) presented the protagonist expressing the emotion that was atypical of the target child (e.g., being happy to see the dog if the mother had reported that the child was scared of dogs). Thus, children had to label emotions nonegocentrically, scoring between 0 and 2 as above (range 0–12).

The three TEC items involved (a) simple causes of emotions, (b) relations between desires and emotions, and (c) knowledge/ignorance and emotion. For (a), children were given five vignettes (e.g., child looking at his/her pet turtle that had just died) and had to label the target emotion by pointing to one of five cartoon faces (happy, sad, angry (‘cross’), scared, all right). For (b), children received two items to assess their understanding of someone's emotional response to a desire being satisfied or unsatisfied (e.g., receiving a drink they liked or hated when they were thirsty). For (c), one item assessed whether children understood the relation between knowledge and emotional response (i.e., a rabbit being unaware of a wolf behind a bush). For each item, children received 1 point for each emotion they labeled correctly, yielding total potential scores between 0 and 10. Including the items on the Denham task and the TEC, internal reliability was adequate, Cronbach's *α*=.66. Children received a total score for performance on the Denham and TEC tasks.

#### Children's behavioral difficulties

Mothers and teachers reported on the children's difficulties at Phase 3 (61 months) using the Strengths and Difficulties Questionnaire (SDQ) (Goodman, 1997). The SDQ includes 25 items rated on a 3‐point scale. Subscales yield scores for externalizing difficulties (total of conduct problems and hyperactivity subscales) and internalizing difficulties (total of emotional symptoms and peer problems subscales), each of which can range between 0 and 20.

Consistent with prior research on reports of externalizing behaviors from multiple sources (Reynolds & Kamphaus, 1992), parent–teacher agreement using correlations was moderate for externalizing behavior (intraclass correlation=.43). Thus, as suggested by Kamphaus and Frick (2002), a simple either/or approach was used, such that each resolved score was calculated as the higher score for each item if the mother and teacher disagreed. Internal reliability of resolved scores for externalizing behaviors was Cronbach's *α*=.81.

#### Children's dysfunctional personality traits

At Phase 4, children reported on CU and impulsive/irresponsibility traits using the Youth Psychopathic Traits Inventory, which was designed to capitalize on the features of psychopathy by phrasing the statements as positive attributes to allow for endorsement of items (e.g., ‘I have the ability not to feel guilt and regret about things that I think other people would feel guilty about’; Andershed, Kerr, Stattin, & Levander, 2002). Fifteen items comprised each scale and were rated on a 4‐point scale, with potential scores for CU and impulsive/irresponsibility both ranging from 15 to 60. These scales have been validated with samples from different countries, showing positive relations with self‐reported conduct problems (Andershed, Gustafson, Kerr, & Stattin, 2002), and emotional deficits in children of similar ages to the ones used here (e.g., 11 years, Wolf & Centifanti, 2014). The internal consistency of the CU and impulsivity/irresponsibility scales was acceptable (*α *= .66; *α *= .73, respectively) and descriptives (see Table 1) were similar to prior research (Mean [*SD*; alpha] of CU in which the atypically developing sample was 36.54 [6.05; *α *= .60], Wolf & Centifanti, 2014). Further, CU and impulsivity/irresponsibility showed no evidence of deviations from normality (Skewness = .41, *z*‐score = 1.67, Kurtosis = .28, *z*‐score = 0.58; Skewness = −.19, *z*‐score = −0.76, Kurtosis = .27, *z*‐score = 0.56, respectively).

**Table 1:** Descriptive statistics and zero‐order correlations among covariates

	*M*	*SD*	1	2	3	4	5	6	7	8	9
1. Gender (1 = female)			–								
2. Socioeconomic Status	33.99	13.99	−.02	–							
3. Maternal Sensitivity	5.64	1.48	−.12	.26***	–						
4. Appropriate Mind‐Related Comments	5.33	3.63	.01	.16*	.34***	–					
5. BPVS	102.34	13.17	−.07	.35***	.25***	.18*	–				
6. Emotion Understanding	34.93	5.78	.09	.33***	.22**	.30***	.54***	–			
7. ToM	2.97	1.75	.17*	.21**	.17*	.24***	.42***	.47***	–		
8. Externalizing	6.83	3.83	−.26***	−.29***	−.16*	−.15	−.22**	−.30***	−.30***	–	
9. CU traits	30.20	5.82	−.28***	−.13	−.06	−.17*	−.30***	−.41***	−.14	.28***	–
10. Impulsive/Irresponsible	30.56	5.81	−.03	−.10	−.02	−.08	−.24***	−.26***	.29***	.27***	.38***

*p < .05; **p < .01; ***p < .001. CU, Callous‐unemotional traits; BPVS, British Picture Vocabulary Scale; ToM, theory of mind.

#### Verbal ability

The British Picture Vocabulary Scale‐II (BPVS) (Dunn, Whetton, & Burley, 1997) was used to assess receptive verbal ability at 51 months. Standardized scores were used as covariates in analyses.

### Data analytic plan

Consistent with our aims, we tested indirect and direct paths from the mothers' appropriate mind‐related comments to CU traits and impulsive/irresponsible traits, controlling for covariates of gender, SES, verbal ability, and maternal sensitivity. The indirect effects of emotion understanding and ToM at 51 months were tested. Mplus 7.2 (Muthén & Muthén, 2012) was used for all analyses (with manifest variables), using bootstrapping of standard errors and confidence intervals to determine the significance of direct and indirect effects (Preacher & Hayes, 2008). Bootstrapping has been recommended when multivariate normality cannot be assumed, typical of small sample sizes. Multivariate normality is especially important when multiple mediators are being included in determining indirect effects in path analysis models using the delta method (Preacher & Hayes, 2008). Bootstrapping was tested at various samples, with no further changes noted after 3000; bootstrapping at 3000 samples was used for all analyses.

We used full information maximum likelihood because we used raw data with some missingness at Phases 3 and 4. The full information maximum likelihood techniques provide less biased estimates than listwise or pairwise deletion (Schafer & Graham, 2002), and are used even when data are not missing at random (Little & Rubin, 2002). Proportions of missing data are examined by a covariance ‘coverage’ provided by Mplus. The minimum coverage is recommended at .10 (Muthén & Muthén, 2012). Coverage ranged from .58 to 1.00 and 156 observations were retained using maximum likelihood estimation.

## Results

### Predictors of CU traits and impulsive/irresponsible traits

Zero‐order correlations are shown in Table 1. In the full sample, we tested whether appropriate mind‐related comments predicted variance in CU traits and impulsive/irresponsible traits via emotion understanding and ToM beyond that predicted by child language abilities, child gender, and SES. Thus, using path analysis in Mplus with continuous data, we regressed these predictors onto CU traits and impulsive/irresponsible traits. This model was fully saturated.

**Figure 1:**
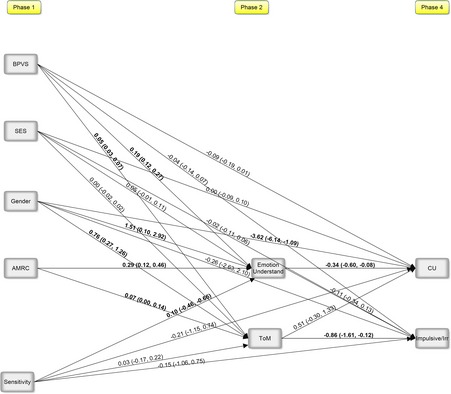
Path analysis predicting CU traits and impulsive/irresponsible traits at10 years of age. Note: Bold significant based on absence of zero in the bootstrapped confidence intervals; Gender (0 = male; 1 = female); CU, callous‐unemotional traits; ToM, theory of mind; AMRC, appropriate mind‐related comments; BPVS, British Picture Vocabulary Scale; SES, socioeconomic status. Indirect effect: AMRC→Emotion Understand→CU: estimate = −0.10, 95% CI: −0.19, −0.004; AMRC→ToM→Impulsive/Irresponsible: estimate = −0.06, 95% CI: −0.14, 0.02; *R* ^2 ^= .34*(Emotion understanding); .23* (ToM); .29* (CU); .17* (Impulsive/Irresponsible)

Unstandardized estimates, interpreted as regression weights, and the associated confidence intervals of all regression paths in the path analysis are presented in Figure 1. Emotion understanding and ToM at 51 months were positively predicted by gender, child language abilities, and appropriate mind‐related comments; thus, higher verbal ability, being female, and having a mother who used more appropriate mind‐related comments predicted better understanding of emotions and ToM. Maternal sensitivity predicted emotion understanding, but not ToM.

As shown in Figure 1, CU traits were predicted by emotion understanding and gender, such that lower levels of CU traits were associated with higher levels of emotion understanding and the child being female. CU traits and impulsive/irresponsible traits were positively related in the model, estimate=8.81, 95% CI: 2.67, 14.96. Emotion understanding and ToM at 51 months were also significantly related, estimate=1.80, 95% CI: 0.77, 2.83. These were moderate effect sizes. ToM significantly predicted impulsive/irresponsible traits. Further, appropriate mind‐related comments significantly predicted CU traits through the indirect effect of emotion understanding, estimate = −0.10, 95% CI: −0.19, −0.004. We used *κ* ^2^ as calculated by R 3.3.0 (R Core Team, 2015) MBESS package version 3.3.3 (Kelley & Lai, 2012), which estimates the effect size of the indirect effect through calculations involving the unstandardized regression coefficients and elements from the covariance matrices (Preacher & Hayes, 2008). *κ* ^2^ reflects the ratio of the obtained indirect effect in relation to the total possible effect attainable. The estimate was .03 with bootstrapped confidence intervals (95%CI= .001, .12), indicating a moderate effect size (Preacher & Hayes, 2008). The indirect effect of appropriate mind‐related comments predicting impulsive/irresponsible traits through ToM was not significant.

### Controlling for effects of externalizing behaviors

**Figure 2:**
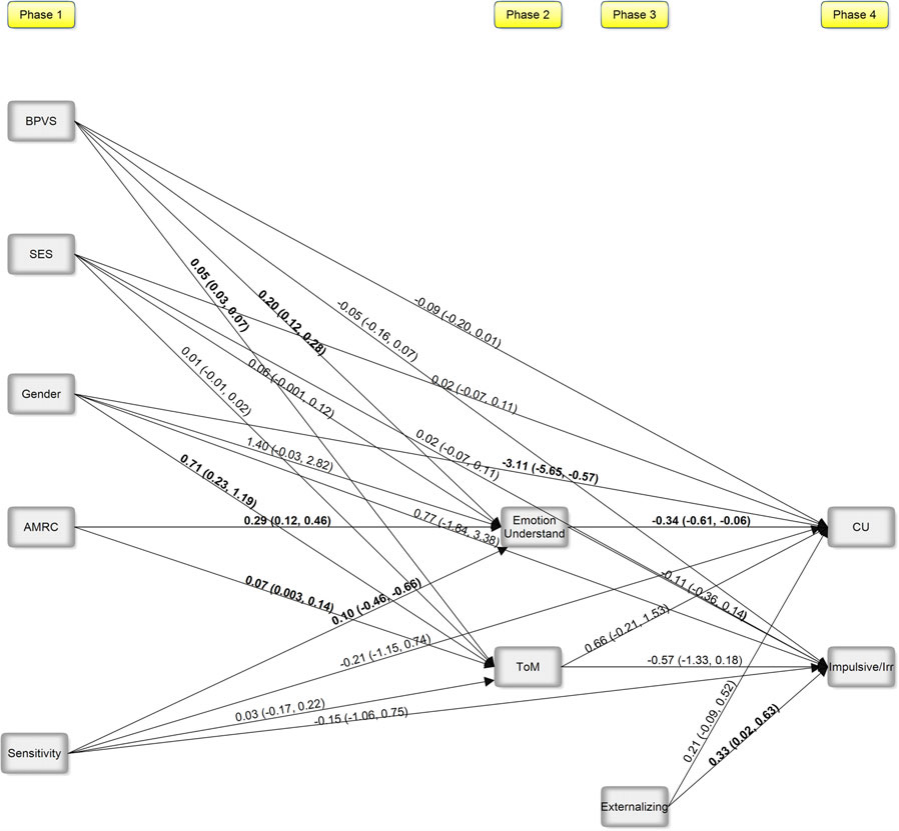
Path analysis predicting CU traits and impulsive/irresponsible traits at 10 years of age, controlling for externalizing behavior. Note: Bold significant based on the absence of zero in the bootstrapped confidence intervals; Gender (0 = male; 1 = female); CU, callous‐unemotional traits; ToM, theory of mind; AMRC, appropriate mind‐related comments; BPVS, British Picture Vocabulary Scale; SES, socioeconomic status. Indirect effect: AMRC→Emotion Understand→CU: estimate = −0.10, 95% CI: −0.19, −0.001; *R* ^2 ^= .36*(Emotion understanding); .24* (ToM); .29* (CU); .15* (Impulsive/Irresponsible)

For this path analysis, 206 observations were retained. Unstandardized estimates and the associated confidence intervals of all regression paths are presented in Figure 2. The model predicting CU traits remained similar. Controlling for externalizing behavior, appropriate mind‐related comments continued to predict CU traits through the indirect effect of emotion understanding. Externalizing behaviors predicted impulsive/irresponsible traits and ToM ceased to be a significant predictor with the externalizing behaviors controlled.

## Discussion

This is the first study to show that early parent–infant interaction predicts CU traits in preadolescence. Early appropriate mind‐related comments were related to lower CU traits a decade later via increases in emotion understanding at age 4. Although appropriate mind‐related comments predicted ToM, which in turn predicted impulsivity/irresponsibility, the indirect effect from mind‐mindedness to impulsivity was nonsignificant. When including early externalizing behaviors, ToM no longer predicted impulsivity/irresponsibility. That is, our findings suggest the variance shared with the other constructs and externalizing behaviors explained a proportion of the association between ToM and impulsivity/irresponsibility. Impulsivity/irresponsibility may be closely linked with externalizing symptoms, as measured by the SDQ (which includes hyperactivity/conduct problems). However, the zero‐order correlations showed externalizing behaviors to be as strongly related to CU traits as to impulsivity/irresponsibility, yet the indirect effect from appropriate mind‐mindedness to CU remained significant when controlling for externalizing behaviors. In contrast, no direct or indirect effects were observed for relations between early maternal sensitivity and CU traits or impulsivity/irresponsibility at age 10. Both general sensitivity and appropriate mind‐related comments appear to ‘scaffold’ a richness in children's understanding of emotions, but only appropriate mind‐related comments led to lower levels of CU traits through this greater understanding.

Research shows that, compared to treatment‐as‐usual, training children with high CU traits on perception and interpretation of human emotions improved parent‐reported child empathy (Dadds, Cauchi, Wimalaweera, Hawes, & Brennan, 2012). There is also converging evidence for the positive impact of appropriately and mutually responsive parental relationships, rather than punitive discipline techniques, on the behavior of children with high CU traits (e.g., Hawes & Dadds, 2005; Kochanska, Kim, Boldt, & Yoon, 2013). Some researchers have called for parenting behaviors to be assessed as early as possible to capitalize on their beneficial effects on the child's early socioemotional development (Boivin et al., 2005). Targeting the early parent–child relationship may be a way to prevent CU traits and their associated problem behaviors, as CU traits have been found to predict increasing externalizing behaviors over time (Waller, Hyde, Grabell, Alves, & Olson, 2015), and now constitute diagnostic features related to a more severe and early‐onset form of conduct disorder (American Psychiatric Association, 2013). Our results suggest early infancy may be an important target for intervention, and highlight the importance of focusing on parental mind‐mindedness rather than general sensitivity. Interventions that focus on promoting mind‐mindedness by drawing parents' attention to their infants' internal states and encouraging them to comment appropriately on what they might be thinking or feeling may thus prove effective.

The present findings must be interpreted in light of limitations. Because we collected measures of CU traits and impulsivity/irresponsibility at the last phase of the study, we cannot say whether there were reciprocal transactions between the CU traits and parenting and children's emotion understanding. Also, we did not include measures of emotion understanding and ToM at the last phase, which precludes controlling for the stability of emotion understanding and ToM. Longitudinal studies are one way to show causality, but the processes may unfold with reciprocal transactions between parents and children. It would thus have been beneficial to obtain parent‐report on children's CU traits in addition to the children's self‐report, although our use of separate reporters for the different measures ensured minimal inflation of effects from shared‐method variance. Finally, we did not measure mothers' CU traits which may arguably be associated with child CU traits given the substantial shared heritability of these traits (Viding, Jones, Frick, Moffitt, & Plomin, 2008). Children with high CU traits may have had mothers who were similarly high on CU, and maternal CU‐related traits could have led mothers failing to use appropriate mind‐related comments. Future research should investigate how maternal CU traits relate to mind‐mindedness. It is interesting, however, that the indirect effect we observed provides a possible mechanism by which commenting appropriately about mental states accounts for child CU traits: through children's ability to read others' emotions.

## Conclusion

This study fills a crucial gap in knowledge regarding early relationship indicators of those with CU traits and other traits related to externalizing behaviors. Maternal mind‐mindedness promotes children's emotion understanding and may cue children to considering other people's emotions in a similarly attuned way to their parents. Family interventions may be one way to improve parent–child relationships and communication. The present findings suggest that parents who remark appropriately on their infants' mental states may help them to understand emotions and may mold an empathic understanding of others, thereby preventing CU traits.

**Key points**

- Problems in understanding other people's mental states may relate to distinct personality traits.
- However, very little work has investigated the mechanisms early in a child's development that might account for these empathic deficits that characterize dysfunctional personality traits (i.e., impulsivity and callous‐unemotional traits).
- In a longitudinal cohort, we examined whether ToM and empathy skills might differentially relate to impulsivity and callous‐unemotional traits.
- We show early appropriate mind‐related comments were related to lower CU traits a decade later via increases in emotion understanding.
- These findings suggest parents who remark appropriately about the child's mental states may help the child to understand emotions and may mold an empathic understanding of others, thereby preventing callous‐unemotional traits.

## References

[jcpp12445-bib-0001] Ainsworth, M.D.S. , Bell, S.M. , & Stayton, D.J. (1974). Infant mother attachment and social development: Socialisation as a product of reciprocal responsiveness to signals In Richards M.P.M. (Ed.), The integration of a child into a social world (pp. 99–136). New York: Cambridge University Press.

[jcpp12445-bib-0002] American Psychiatric Association . (2013). Diagnostic and statistical manual of mental disorders, 5th edition (DSM‐5). American Psychiatric Association: Washington, DC.

[jcpp12445-bib-0003] Andershed, H.A. , Gustafson, S.B. , Kerr, M. , & Stattin, H. (2002). The usefulness of self‐reported psychopathy‐like traits in the study of antisocial behaviour among non‐referred adolescents. European Journal of Personality, 16, 383–402.

[jcpp12445-bib-0004] Andershed, H.A. , Kerr, M. , Stattin, H. , & Levander, S. (2002). Psychopathic traits in non‐referred youths: A new assessment tool In Blauuw E. & Sheridan L. (Eds.), Psychopaths: Current International perspectives (pp. 131–158). The Hague: Elsevier.

[jcpp12445-bib-0005] Astington, J.W. , & Jenkins, J.M. (1999). A longitudinal study of the relation between language and theory‐of‐mind development. Developmental Psychology, 35, 1311–1320. 10493656 10.1037//0012-1649.35.5.1311

[jcpp12445-bib-0006] Bedford, R. , Pickles, A. , Sharp, H. , Wright, N. , & Hill, J. (2015). Reduced face preference in infancy: A developmental precursor to callous‐unemotional traits? Biological Psychiatry, 78, 144–150. 25526972 10.1016/j.biopsych.2014.09.022 PMC4510143

[jcpp12445-bib-0007] Boivin, M. , Pérusse, D. , Dionne, G. , Saysset, V. , Zoccolillo, M. , Tarabulsy, G.M. , … & Tremblay, R.E. (2005). The genetic‐environmental etiology of parents' perceptions and self‐assessed behaviours toward their 5‐month‐old infants in a large twin and singleton sample. Journal of Child Psychology and Psychiatry and Allied Disciplines, 46, 612–630. 10.1111/j.1469-7610.2004.00375.x 15877767

[jcpp12445-bib-0008] Dadds, M.R. , Cauchi, A.J. , Wimalaweera, S. , Hawes, D.J. , & Brennan, J. (2012). Outcomes, moderators, and mediators of empathic‐emotion recognition training for complex conduct problems in childhood. Psychiatry Research, 199, 201–207. 22703720 10.1016/j.psychres.2012.04.033

[jcpp12445-bib-0009] Dadds, M.R. , Hawes, D.J. , Frost, A.D. , Vassallo, S. , Bunn, P. , Hunter, K. , & Merz, S. (2009). Learning to ‘talk the talk: The relationship of psychopathic traits to deficits in empathy across childhood. Journal of Child Psychology and Psychiatry and Allied Disciplines, 50, 599–606. 10.1111/j.1469-7610.2008.02058.x 19445007

[jcpp12445-bib-0010] Dawel, A. , O'Kearney, R. , McKone, E. , & Palermo, R. (2012). Not just fear and sadness: Meta‐analytic evidence of pervasive emotion recognition deficits for facial and vocal expressions in psychopathy. Neuroscience and Biobehavioral Reviews, 36, 2288–2304. 22944264 10.1016/j.neubiorev.2012.08.006

[jcpp12445-bib-0011] Denham, S.A. (1986). Social cognition, prosocial behavior, and emotion in preschoolers: Contextual validation. Child Development, 57, 194–201.

[jcpp12445-bib-0012] Dunn, L.M. , Whetton, C. , & Burley, J. (1997). British picture vocabulary scale (2nd edn). Windsor, UK: NFER‐Nelson.

[jcpp12445-bib-0013] Fahie, C.M. , & Symons, D.K. (2003). Executive functioning and theory of mind in children clinically referred for attention and behavior problems. Journal of Applied Developmental Psychology, 24, 51–73.

[jcpp12445-bib-0014] Goodman, R. (1997). The Strengths and Difficulties Questionnaire: A research note. Journal of Child Psychology and Psychiatry, 38, 581–586. 9255702 10.1111/j.1469-7610.1997.tb01545.x

[jcpp12445-bib-0015] Hawes, D.J. , & Dadds, M.R. (2005). The treatment of conduct problems in children with callous‐unemotional traits. Journal of Consulting and Clinical Psychology, 73, 737–741. 16173862 10.1037/0022-006X.73.4.737

[jcpp12445-bib-0016] Hollingshead, A . (1975). Four factor index of social status. Yale Journal of Sociology, 8, 21–52. Retrieved from http://elsinore.cis.yale.edu/sociology/yjs/yjs_fall_2011.pdf#page=21 [last accessed 28 October 2014].

[jcpp12445-bib-0017] Kamphaus, R.W. , & Frick, P.J. (2002). Clinical assessment of children's personality and behavior (2nd edn) New York: Allyn & Bacon.

[jcpp12445-bib-0018] Kelley, K. , & Lai, K . (2012). MBESS: MBESSR. R package version 3.3.3. Retrieved from http://cran.r-project.org/package=MBESS [last accessed 28 October 2014].

[jcpp12445-bib-0019] Kochanska, G. (1993). Toward a synthesis of parental socialization and child temperament in early development of conscience. Child Development, 64, 325–347.

[jcpp12445-bib-0500] Kochanska, G. , Kim, S. , Boldt, L.J. , & Yoon, J.E. (2013). Children's callous‐unemotional traits moderate links between their positive relationships with parents at preschool age and externalizing behavior problems at early school age. Journal of Child Psychology and Psychiatry, 54, 1251–1260. 23639120 10.1111/jcpp.12084 PMC3740026

[jcpp12445-bib-0020] Laranjo, J. , Bernier, A. , Meins, E. , & Carlson, S.M. (2010). Early manifestations of children's theory of mind: The roles of maternal mind‐mindedness and infant security of attachment. Infancy, 15, 300–323. 10.1111/j.1532-7078.2009.00014.x 32693541

[jcpp12445-bib-0021] Little, R.J.A. , & Rubin, D.B. (2002). Statistical analysis with missing data, 2nd ed. Hoboken, NJ: John Wiley & Sons Inc.

[jcpp12445-bib-0022] Marsee, M.A. , & Frick, P.J. (2007). Exploring the cognitive and emotional correlates to proactive and reactive aggression in a sample of detained girls. Journal of Abnormal Child Psychology, 35, 969–981. 17636437 10.1007/s10802-007-9147-y

[jcpp12445-bib-0023] Marsee, M.A. , Frick, P.J. , Barry, C.T. , Kimonis, E.R. , Muñoz Centifanti, L.C. , & Aucoin, K.J. (2014). Profiles of the forms and functions of self‐reported aggression in three adolescent samples. Development and Psychopathology, 26, 705–720. 25047293 10.1017/S0954579414000339

[jcpp12445-bib-0024] Marsh, A.A. , & Blair, R.J.R. (2008). Deficits in facial affect recognition among antisocial populations: A meta‐analysis. Neuroscience and Biobehavioral Reviews, 32, 454–465. 17915324 10.1016/j.neubiorev.2007.08.003 PMC2255599

[jcpp12445-bib-0025] Meins, E. , Centifanti, L.C.M. , Fernyhough, C. , & Fishburn, S. (2013). Maternal mind‐mindedness and children's behavioral difficulties: Mitigating the impact of low socioeconomic status. Journal of Abnormal Child Psychology, 41, 543–553. 23299554 10.1007/s10802-012-9699-3

[jcpp12445-bib-0026] Meins, E. , Fernyhough, C. , Arnott, B. , Leekam, S.R. , & de Rosnay, M. (2013). Mind‐mindedness and theory of mind: Mediating roles of language and perspectival symbolic play. Child Development, 84, 1777–1790. 23432622 10.1111/cdev.12061

[jcpp12445-bib-0027] Meins, E. , Fernyhough, C. , Wainwright, R. , Das Gupta, M. , Fradley, E. , & Tuckey, M. (2002). Maternal mind‐mindedness and attachment security as predictors of theory of mind understanding. Child Development, 73, 1715–1726. 12487489 10.1111/1467-8624.00501

[jcpp12445-bib-0028] Muñoz, L.C. (2009). Callous‐unemotional traits are related to combined deficits in recognizing afraid faces and body poses. Journal of the American Academy of Child and Adolescent Psychiatry, 48, 554–562. 19318989 10.1097/CHI.0b013e31819c2419

[jcpp12445-bib-0029] Muñoz, L.C. , & Frick, P.J. (2012). Callous‐unemotional traits and their implication for understanding and treating aggressive and violent youths. Criminal Justice and Behavior, 39, 794–813.

[jcpp12445-bib-0030] Muñoz, L.C. , Qualter, P. , & Padgett, G. (2011). Empathy and bullying: Exploring the influence of callous‐unemotional traits. Child Psychiatry and Human Development, 42, 183–196. 20886285 10.1007/s10578-010-0206-1

[jcpp12445-bib-0031] Muthén, L. , & Muthén, B. (2012). Mplus user's guide, 5th ed. Los Angeles: Author.

[jcpp12445-bib-0032] O'Nions, E. , Sebastian, C.L. , McCrory, E. , Chantiluke, K. , Happé, F. , & Viding, E. (2014). Neural bases of theory of mind in children with autism spectrum disorders and children with conduct problems and callous‐unemotional traits. Developmental Science, 17, 786–796. 24636205 10.1111/desc.12167 PMC4316185

[jcpp12445-bib-0033] Perner, J. , Kain, W. , & Barchfeld, P. (2002). Executive control and higher‐order theory of mind in children at risk of ADHD. Infant and Child Development, 11, 141–158.

[jcpp12445-bib-0034] Pons, F. , Harris, P.L. , & de Rosnay, M . (2004). Emotion comprehension between 3 and 11 years: Developmental periods and hierarchical organization. European Journal of Developmental Psychology, 1, 127–152.

[jcpp12445-bib-0035] Preacher, K.J. , & Hayes, A.F. (2008). Asymptotic and resampling strategies for assessing and comparing indirect effects in multiple mediator models. Behavior Research Methods, 40, 879–891. 18697684 10.3758/brm.40.3.879

[jcpp12445-bib-0036] R Core Team . (2015). R: A language and environment for statistical computing. Vienna, Austria: R Foundation for Statistical Computing Retrieved from http://www.r-project.org/ [last accessed 28 October 2014].

[jcpp12445-bib-0037] Reynolds, C.R. , & Kamphaus, R.W.R. (1992). Behavior Assessment System for Children. Circle Pines, MN: AGS Publishing.

[jcpp12445-bib-0038] Schafer, J.L. , & Graham, J.W. (2002). Missing data: Our view of the state of the art. Psychological Methods, 7, 147–177. 12090408

[jcpp12445-bib-0039] Sharp, C. , Vanwoerden, S. , Van Baardewijk, Y. , Tackett, J.L. , & Stegge, H. (2015). Callous‐unemotional traits are associated with deficits in recognizing complex emotions in preadolescent children. Journal of Personality Disorders, 29, 347–359. 25248014 10.1521/pedi_2014_28_161

[jcpp12445-bib-0040] Viding, E. , Jones, A.P. , Frick, P.J. , Moffitt, T.E. , & Plomin, R. (2008). Heritability of antisocial behaviour at 9: Do callous‐unemotional traits matter? Developmental Science, 11, 17–22. 18171362 10.1111/j.1467-7687.2007.00648.x

[jcpp12445-bib-0041] Viding, E. , McCrory, E. , & Seara‐Cardoso, A. (2014). Psychopathy. Current Biology, 24, R871–R874. 25247365 10.1016/j.cub.2014.06.055

[jcpp12445-bib-0042] Völlm, B.A. , Taylor, A.N.W. , Richardson, P. , Corcoran, R. , Stirling, J. , McKie, S. , … & Elliott, R. (2006). Neuronal correlates of theory of mind and empathy: A functional magnetic resonance imaging study in a nonverbal task. NeuroImage, 29, 90–98. 16122944 10.1016/j.neuroimage.2005.07.022

[jcpp12445-bib-0043] Waller, R. , Hyde, L.W. , Grabell, A.S. , Alves, M.L. , & Olson, S.L. (2015). Differential associations of early callous‐unemotional, oppositional, and ADHD behaviors: Multiple domains within early‐starting conduct problems? Journal of Child Psychology and Psychiatry, and Allied Disciplines, 56, 657–666. 10.1111/jcpp.12326 PMC4937618 25251938

[jcpp12445-bib-0044] Wellman, H.M. , & Liu, D. (2004). Scaling of theory‐of‐mind tasks. Child Development, 75, 523–541. 15056204 10.1111/j.1467-8624.2004.00691.x

[jcpp12445-bib-0045] Wolf, S. , & Centifanti, L.C.M . (2014). Recognition of pain as another deficit in young males with high callous‐unemotional traits. Child Psychiatry and Human Development, 45, 422–432. 24276392 10.1007/s10578-013-0412-8

